# Alterations of Mitochondrial Biology in the Oral Mucosa of Chilean Children with Autism Spectrum Disorder (ASD)

**DOI:** 10.3390/cells8040367

**Published:** 2019-04-23

**Authors:** Manuel Carrasco, Celia Salazar, William Tiznado, Lina María Ruiz

**Affiliations:** 1 Instituto de Ciencias Biomédicas, Facultad Ciencias de la Salud, Universidad Autónoma de Chile, Santiago 8910060, Chile; flgo.manuelcarrasco@gmail.com (M.C.); celia.salazar@uautonoma.cl (C.S.); 2 ASPAUT, Escuela Especial Magdalena Ávalos Cruz, San Miguel, Santiago 8900044, Chile; 3 Computational and Theoretical Chemistry Group, Departamento de Ciencias Químicas, Facultad de Ciencias Exactas, Universidad Andres Bello (UNAB), Santiago 8370251, Chile; wtiznado@unab.cl

**Keywords:** autism, ASD, mitochondrial DNA, oral mucosa, oxidative stress, gene expression

## Abstract

Autistic Spectrum Disorder (ASD) is characterized by the impairment of socio-communicative skills and the presence of restricted and stereotyped behavior patterns. Recent researches have revealed the influence of mitochondrial physiology on the development of ASD. Several research groups have identified defects in respiratory complexes, coenzyme-Q10 deficiency, increased oxidative damage, decreased of superoxide dismutase (SOD2). A study on the influence of mitochondrial physiology on the development of ASD can provide new alternatives and challenges. That is why we set ourselves the general objective to initiate studies of mitochondrial physiology in Chilean children with ASD. A sample of oral mucosa was collected in a group of 12 children diagnosed with ASD and 12 children without ASD. In children with ASD, we found a significant increase in mitochondrial DNA levels. Likewise, in these children, an increase in the protein oxidation was observed. Finally, a downward trend in the expression of the *HIGD2A* and *SOD2* genes was observed, while *DRP1*, *FIS1*, *MFN1*, *MFN2*, and *OPA1* gene expression show an upward trend. The increment of mitochondrial DNA, high oxidative stress, and high expression of the *MFN2* gene could help as a scanner of the mitochondrial function in children with ASD.

## 1. Introduction

The Autism Spectrum Disorder (ASD) is an alteration of the neurodevelopment with an impact throughout life that present impediments in the social interaction, communication, imagination, and repetitive behaviors, that could affect 1 to 2% of children [1,2,3,4,5,6,7,8,9]. Early diagnosis and intervention allow a better outlook since the progress is significant in linguistic development, social ability, and the possibility of higher behavioral adaptation [10,11]. Therefore, it is essential to advance in the study of molecular mechanism of ASD. 

The ASD has a multifactorial etiology in which the oxidative stress is one of the pathology mechanisms widely described [12]. In the last years, many investigations have focused on the study of mitochondrial dysfunction in children with ASD [13,14,15,16,17]. The first studies that associated the alteration of bioenergetics metabolism with the ASD reported high levels of lactic acid, pyruvate, and serotonin [13]. Subsequently, defects were identified in every I, II, III, IV, and V respiratory complex, deficiency of coenzyme Q_10_ (CoQ10), defects in mitochondrial genes and nuclear genes [9,14,15,16].

In ASD, the mitochondrial dysfunction has been related to a deficit in the cellular bioenergetics that entails synaptic defects and a reduction in the neurotransmitter’s liberation. This deficit in the mitochondrial energy production in individuals with ASD is mainly associated with deficiencies in complex I and complex III in the cerebellum, prefrontal cortex, and temporal area [17]. In the cerebral cortex, on lateral temporal lobe BA21, alterations in the respiratory complex expressions of post-Morten individuals with ASD, a decrease of enzymatic activity of complex I and IV, the increase of oxidative damage in the DNA, and decrease of mitochondrial antioxidant enzyme manganese superoxide dismutase (SOD2) were found [18]. Regarding the SOD2 dysfunction, the Ala16Val polymorphism (rs4880) associated with a reduction in the enzymatic activity and increase in the oxidative stress [19] has been widely studied. The altered cellular localization of SOD2 could be part of the neurodegeneration process that occurs in ASD. This Ala16Val polymorphism has been briefly described in children with ASD [20]. 

Additionally, autism has been reported alterations in the level of mitochondrial DNA (mtDNA) [14,16,21,22]. Children with ASD were more susceptible to mitochondrial dysfunction, increase in replication of mtDNA, and deletions of mtDNA in with children with a typical development [14,21], although other investigators did not observe a variation in the sequence or levels of mtDNA in ASD [18,23].

Moreover, in the cerebral cortex, on the lateral temporal lobe BA21 of post-Morten individuals with ASD an alteration in the mitochondrial dynamics with high levels of the proteins implicated in the mitochondrial fission (FIS1 and DRP1) and a decrease in the levels of the proteins implicated in the mitochondrial fusion (MFN1, MFN2, OPA1) [18] were identified. 

However, an adequate understanding of the mitochondrial dysfunction in autism is difficult because the evaluation of mitochondrial function requires an invasive biopsy that can result in more complications for children with autism. The use of noninvasive procedures, such as obtaining oral mucosa, offer an alternative for study the mitochondrial dysfunction in autism. The oral mucosa derives from embryonic ectoderm, as well as the central nervous system [24], making oral mucosa a relevant tissue in the study of autism. Studies conducted by Goldenthal et al. validated the oral mucosa in the study of mitochondrial diseases [25]. Since then, several studies have taken advantage of the oral mucosa evaluation in the study of mitochondrial dysfunction in autism [26,27]. Goldenthal et al. (2015) showed extensive abnormalities in the activity of mitochondrial respiratory complex I and IV in a buccal swab analysis of ASD children. These authors proposed a noninvasive biomarker to asses mitochondrial function in ASD patients [26] for the first time. Studies evaluating treatments for children with ASD who have mitochondrial dysfunction are minimal, but some report improvements by supplementing children with carnitine, Coenzyme Q10, and B vitamins [15]. Frye et al. (2013) pointed to the importance of identifying children with ASD who have mitochondrial dysfunction, since they may respond differently to specific treatments due to their specific metabolic profile [28]. Recently, Delhey et al. (2017) showed promising results administering specific mitochondrial supplements, such as fatty acid, folate, B12, which may increase the activity of complex I, complex IV, and citrate synthase [29]. Remarkably, the treatment of cell lines from ASD patients with butyrate, a ubiquitous short-chain fatty acid, enhances mitochondrial function during oxidative stress [30]. 

Interestingly, the mitochondrial protein HIG2A, is a mediator of the respirosome assembly and the supramolecular association of the I, III, and IV respiratory complex [31], allowing an increase of electron transfer, substrate canalization, catalytic potentiation, protein complex stability, and a decrease of oxidative stress [32]. In addition, we show that changes in oxygen concentration, glucose availability, and cell cycle regulate *HIGD2A* expression. Alterations in *HIGD2A* expression are associated with changes in mitochondrial physiology [33]. We hypothesize that the analysis of gene expression that encodes for *HIGD2A*, *SOD2*, *DRP1*, *FIS1*, *MFN1*, *MFN2*, and *OPA1* in children with ASD could help as a scanner of the mitochondrial function.

A better understanding of the relationship between mitochondrial dysfunction in ASD and oxidative stress, along with other factors, such as respiratory complex remodeling and the mitochondrial dynamics, can be critical for understanding how to approach the treatment of children with ASD. The objective for the present work has initiated the study about some aspects of mitochondrial physiology in the oral mucosa of 6- to 13-year-old Chilean children with ASD. From oral mucosa, we isolated DNA, RNA, and proteins, to evaluate the Ala16Val-SOD2 polymorphism, mitochondrial DNA, the *HIGD2A*, *SOD2*, *DRP1*, *FIS1, MFN1*, *MFN2,* and *OPA1* gene expression. Furthermore, we evaluated the oxidation of total proteins and the expression of proteins of the respiratory complex. Our study reported for the first time a high quantity of mitochondrial DNA in oral mucosa as another biomarker of ASD. Moreover, for the first time, the analyses show higher protein carbonylation and the immunodetection of the respiratory complexes in the buccal mucosa of ASD children. In addition, we showed that mitochondrial dynamics-related genes are more expressed in ASD children.

## 2. Materials and Methods

### 2.1. Experimental Design

The research was a non-experimental, descriptive transactional and non-probabilistic design. The data were collected in a single moment, in a single time. The purpose was to measure and describe variables independently. The focus of the research was quantitative. The data collection to test the hypothesis was based on numerical measurement and statistical analysis to establish patterns of molecular behavior.

### 2.2. Subjects

Cheek swabs were collected from both ASD patients (*n* = 12, 11 males and 1 female) and healthy controls (*n* = 12, 9 males and 3 females). The age of the children ranged from 6 to 13 years who achieved a level of understanding of basic instructions by means of pictograms. The selection of children with ASD was carried out at the Magdalena Ávalos Cruz Special School, ASPAUT, in the San Miguel district. All the children who attend this school are diagnosed by the medical professional with Autism Spectrum Disorder. For the selection of healthy control children, the Monte Olivo School in Puente Alto district was chosen. All the children who attend this school are children who access regular school curriculums and do not have a previous medical diagnosis, without any signs or symptoms of neurological disease. In both selections, informed consent for the different samples was elaborated. The documents were authorized by the Institutional Ethics Committee of the Universidad Autónoma de Chile (Act N° 0135-16).

### 2.3. Oral Sample Preparation

The sample collection process was carried out by a non-invasive procedure, where a sample of oral mucosa was taken. Each child was given 10 mL of sterile saline (0.9% NaCl), indicating that mouthwash should be done by sucking the cheeks and not swallowing the saline solution. The indications were accompanied by pictograms. After the mouthwash, the saline was returned to the container and centrifuged at 6000 rpm for 10 min at 4 °C. The cellular precipitate containing the oral mucosa was resuspended in 1 mL of TRIzol™ Reagent (Invitrogen) and stored at −20 °C until the isolation of RNA, DNA, and proteins.

### 2.4. Isolation of RNA, DNA, and Proteins

From oral mucosa RNA, DNA, and proteins were extracted with TRIzol™ Reagent (Invitrogen), according to the manufacturer´s protocol. The quantification of the quantity and quality of the RNA, DNA, and proteins isolated in each sample was carried out by measuring the absorbance (A) using the Infinite M200 Pro spectrophotometer (TECAN). The quality and integrity of the DNA and RNA were evaluated on 1.5% agarose gels.

### 2.5. Reverse Transcriptase and Quantitative Real-Time PCR (qRT-PCR)

Complementary DNA synthesis (cDNA) was performed from 2 μg of purified RNA using the RevertAid First Strand cDNA Synthesis kit (Thermo Scientific, Waltham, MA, USA) which in addition to the reverse transcriptase (RevertAid M-MulV RT), includes a mixture of oligo (dT) and random hexamer starters optimizing the synthesis of cDNA from RNA. For a final volume of 40 μL per reaction, 2 μg of RNA contained in 20 μL of nuclease-free water, 2 μL random hexamer starter, 2 μL of oligo (dT) was prepared. It was incubated for 5 min at 65 °C, and the following mixture was added; 8 μL Buffer 5×, 2 μL RiboLock RNase Inhibitor, 4 μL mixture of deoxyribonucleotide triphosphates (dNTPs), 2 μL reverse transcriptase. The mixture was homogenized gently and centrifuged briefly. Later in the thermocycler, a 5 min incubation was scheduled at 25 °C followed by an incubation for 60 min at 42 °C and 5 min at 72 °C. The resulting cDNA was stored at −20 °C until used. For the relative quantification of cDNA levels by qPCR, the LightCycler^®^ 96-Real-time PCR system (Roche, Risch-Rotkreuz,, Switzerland) with capacity for 96-well plates was available and the FastStart Essential DNA Green Master (Roche) kit based on the fluorophore SyBR Green. Data are presented as relative gene expression normalized to PPIA gene expression. The following primers were used: Fw-SOD2: 5′-AAGGGAGATGTTACAGCCCAGATA-3′; Rev-SOD2: 5′-TCCAGAAAATGCTATGATTGATATGAC-3′, Fw-PPIA: 5′- GTGGTCTTTGGGAAGGTG-3′, Rev-PPIA: 5′-GGTGATCTTCTTGCTGGTC-3′; Fw-HIGD2A: 5′-GGAGTCCCGATTTTCTCCTG-3′; Rev-HIGD2A: 5′-CGGAGCTTTCAAGGCCAG-3′. Fw-MFN1: 5′-GTTGGAGCGGAGACTTAGCA-3′; Rev-MFN1: 5′-TCCGAGATAGCACCTCACCA-3′; Fw-MFN2: 5′-CGCTTATCCACTTCCCTCCTC-3′; Rev-MFN2: 5′-AGCAGGGACATTGCGCTTC-3′; Fw-OPA1: 5′-GTGCTGCCCGCCTAGAAA-3′; Rev-OPA1: 5′-TGACAGGCACCCGTACTCAGT-3′; Fw-FIS1: 5′-TACGTCCGCGGGTTGCT-3′; Rev-FIS1: 5′-CCAGTTCCTTGGCCTGGTT-3′; Fw-DRP1: 5′-TGGGCGCCGACATCA-3′; Rev-DRP1: 5′-GCTCTGCGTTCCCACTACGA-3′.

### 2.6. qPCR of mtDNA Levels

For the Evaluation of mtDNA, qPCR was performed as described earlier [34,35]. From the total DNA, mtDNA was amplified and quantified by real-time PCR (qPCR) using the following specific primers: mitochondrial gene tRNA-Leu (UUU) Fw: CACCCAAGAAC AGGGTTTGT, Rev: TGGCCATGGGTATGTTGTTA; nuclear gene B2-microglobulin Fw: TGCTGTCTCCATGTTTGATGTATCT, Rev: TCTCTGCTCCCCACCTCTAAGT [34,35].

### 2.7. Tetra-Primer Amplification-Refractory Mutation System (ARMS)-PCR

Genotyping of the Ala16Val-SOD2 SNP polymorphism was performed by PCR using the DNA from the oral mucosa sample and a "Tetra-Primer ARMS-PCR" assay [36]. For this, the following primers were used: ARMS-SOD2-F1 5′-CACCAGCACTAGCAGCATGT-3′; ARMS-SOD2-F2 5′-GCAGGCAGCTGGCTaCGGT-3′; ARMS-SOD2-R1 5′-ACGCCTCCTGGTACTTCTCC-3′; ARMS-SOD2-R2 5′-CCTGGAGCCCAGATACCCtAAAG-3′. The PCR product was analyzed on 1.5% agarose gels for the genotypes (Ala-Val [AV], Ala-Ala [AA], Val-Val [VV]) [36].

### 2.8. Western Blot

For the immunodetection of respiratory complexes, 20 µg of total protein samples from oral mucosa were fractionated in 12% polyacrylamide gel with running buffer: twenty-five millimolar Tris, 190 mM glycine, 0.1% SDS, pH 8.3 was transferred onto a polyvinyl difluoride (PVDF) membrane using a wet transfer tank with the transfer buffer: twenty-five millimolar Tris, 190 mM glycine, 20% methanol, pH 8.3. The PVDF membrane was blocked for 1 h with 3% bovine serum albumin (BSA) in Tris-buffered saline with Tween 20 (TBST) buffer; 20 mM Tris pH 7.5, 150 mM NaCl, 0.1% Tween 20, at room temperature. Then, the PVDF membrane was incubated overnight at 4 °C with MitoProfile^®^ Total Oxidative Phosphorylation (OXPHOS) Human Western Blot (WB) Antibody Cocktail (ab110411 Abcam) used at 1/200 dilution in 1% BSA in TBST. The blot was rinsed three times for 5 min with TBST buffer. Primary antibodies were detected with the secondary antibodies: Horseradish peroxidase (HRP)-conjugated anti-mouse Immunoglobulin G (IgG) used at 1/300 in 1% BSA in TBST for 1 h at room temperature and the Chemiluminescent Western Blot Detection (ThermoFisher Scientific).

### 2.9. Protein Oxidation Detection

Total protein oxidation was measured with the Oxyblot Detection Kit (Millipore) according to the manufacturer’s instructions. This measurement is based on the detection of carbonyl groups that are introduced into protein side chains when proteins are exposed to oxidative stress [37]. Briefly, total protein levels were quantified using the Bradford assay and using bovine serum albumin as a standard. The carbonyl groups in the protein side chains are derivatized to 2,4-dinitrophenylhydrazone (DNP-hydrazone) by reaction with 2,4-dinitrophenylhydrazine (DNPH). The protein derivatization reaction was performed with 15 µg of total protein. The DNP-derivatized proteins samples were fractionated in 12% polyacrylamide gel electrophoresis (PAGE) and then transferred onto a polyvinyl difluoride membrane using a wet transfer tank.

### 2.10. Statistical Analysis

Statistical analysis was performed with GraphPad Prism 6 software (GraphPAd Software, Inc., La Jolla, CA, USA). To compare the results of two variables that participated in the same experiment, it was analyzed by means of an unpaired student T-test. For the statistical analysis of the polymorphism the Chi-square test was used to evaluate the differential hypothesis between the two categorical variables ASD and HC, a contingency table was used where each variable was divided into different categories (A/A, A/V, and V/V alleles). Subsequently, the expected frequencies were calculated.

## 3. Results

### 3.1. Subjects Clinical Data and Demographic Information

The age of the 12 ASD children, one female, and 11 males ranged from 7 to 12 years, with an average of 9.6 ± 1.9 years. In Table 1 presents the clinical data and demographic information of the children with ASD. The different therapies mentioned in Table 1, behavioral, communication, and sensory, are focused on Behavioral Programs that allow, through cognitive/behavioral strategies, the modification of maladaptive and disruptive behaviors with sensory and communicative nuances, giving greater comfort to the child and the family in activities of daily life.

### 3.2. Oral Mucosa mtDNA Levels in Chilean Children with ASD

**Figure 1:**
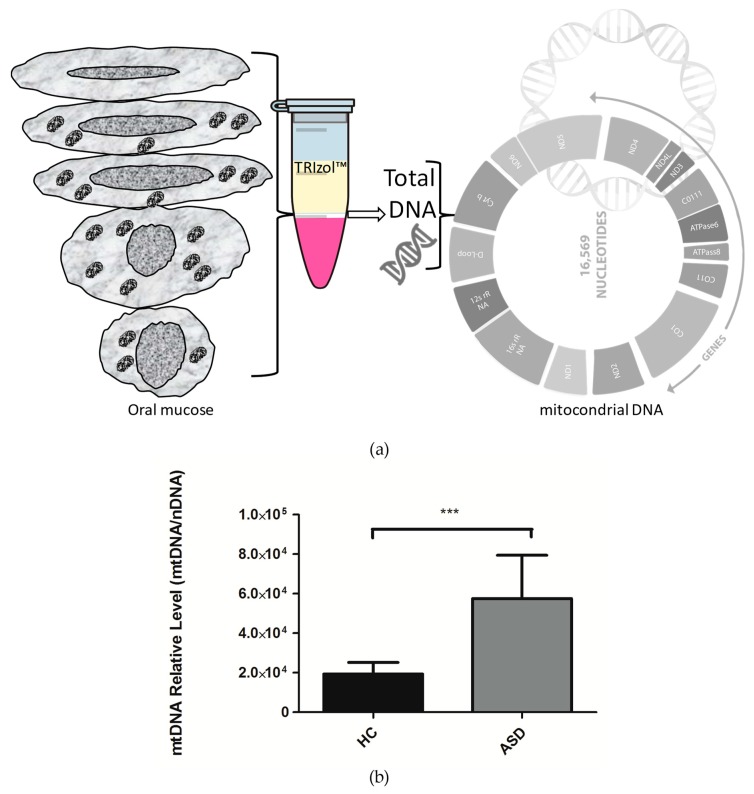
The relative levels of mitochondrial DNA (mtDNA) in children with Autistic Spectrum Disorder (ASD) and Healthy Controls (HC). (**a**) Total DNA was isolated from oral mucose cells, total DNA was used for the analysis of mitochondrial DNA. (**b**) For mitochondrial DNA quantification, the mitochondrial gene tRNA- Leu (UUU) was amplified by qPCR, and the nuclear gene B2-microglobulim amplification was used for the normalization. Each bar graph represents the mean ± SEM, *n* = 12 ASD and 12 HC children, analyzed by unpaired t-test (*P* < 0.05) and producing a *P* value = 0.0006 (***).

Mitochondrial DNA copies are a very common marker in mitochondrial dysfunction, and alterations in mtDNA levels have been reported in autism [14,21]. However, there is no consensus on the different findings obtained by various research groups. Therefore, we set out to explore the levels of mtDNA in Chilean children with ASD. For this, extraction of total DNA from the oral mucosa was performed, and by qPCR, relative quantification of the mtDNA was carried out with a nuclear gene as a gene reference. Our results show that there is a significant increase in the mtDNA levels in the oral mucosa of Chilean children with ASD (Figure 1). These results are consistent with those obtained by the research from other groups that indicate an increase in mtDNA copies and mtDNA deletions in children with ASD, which is correlated with mitochondrial dysfunction [14,21]. However, other groups have not been able to identify differences in mtDNA levels in ASD [18,23]. The increase in mtDNA levels could account for a compensatory mechanism to strengthen the different mitochondrial functions that govern cellular bioenergetics [14]. In addition, this condition is suggestive of alterations in the mitochondrial DNA replisome, where different proteins are involved than those used for nuclear DNA replication; the T7-Like Mitochondrial DNA helicase (TWINKLE) and the DNA Polymerase Gamma (POLγ), among others [38,39].

### 3.3. Oral Mucosa Total Protein Oxidation Levels and Western Blotting Analysis of Respiratory Complexes in Chilean Children with ASD

**Figure 2:**
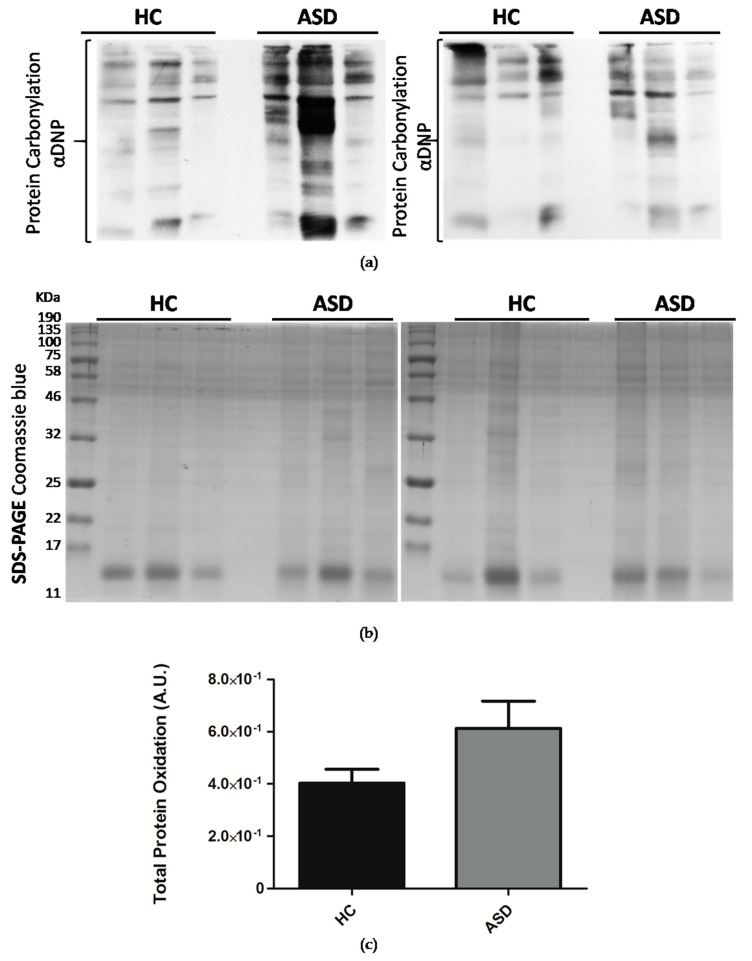
Oral mucosa total protein oxidation levels in Chilean children with ASD. (**a**) Total proteins were isolated from oral mucosa cells by TRIzol™ reagent (Invitrogen) and fractionated by SDS-PAGE (sodium dodecyl sulfate-polyacrylamide gel electrophoresis). Protein carbonylation was detected with the Oxyblot Protein Detection Kit. αDNP: Anti-2,4-dinitrophenylhydrazone antibody. (**b**) SDS-PAGE Coomassie blue staining of SDS-PAGE of derivatized proteins. (**c**) Densitometry quantification of protein carbonylation was made with the ImageJ software. Carbonylation of proteins was normalized by Coomassie blue staining. Each bar represents the mean ± SEM, analyzed by unpaired t-test. Healthy controls (HC), *n* = 11 and ASD children, *n* = 11.

**Figure 3:**
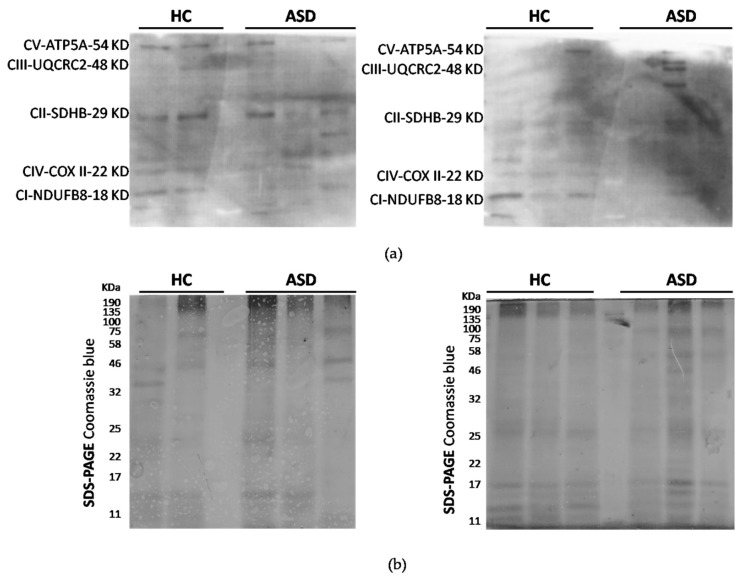
Protein expression analysis of mitochondrial respiratory complexes. (**a**) Oxidative Phosphorylation (OXPHOS) protein expression total proteins were obtained from oral mucosa and fractionated by SDS-PAGE. Complex I (CI), complex II (CII), complex III (CIII), complex IV (CIV) and ATP synthase (CV) were detected by immunoblots. (**b**) SDS-PAGE Coomassie blue staining was used as a loading control. *n* = 6 ASD and 5 HC children.

ASD has enhanced reactive oxygen species (ROS) generation due to alterations in the respiratory chain activities. Total protein oxidation in the oral mucosa of children with ASD was higher than healthy control children (Figure 2). In addition, the protein expression of respiratory complexes was assessed by immunoblot. However, the protein samples were depleted, so only five samples from the control children and six samples from the ASD children were analyzed. The manufacturers of MitoProfile^®^ Total OXPHOS Human WB Antibody Cocktail (ab110411 Abcam) recommended to perform the immunodetection test of the respiratory complexes with 50 µg of protein, but we could only load 20 µg, for this reason, the quality of the immunoblot is low. Although, this result confirms the possibility of detecting proteins from respiratory complexes from samples of the oral mucosa, it is necessary to perform more analyzes that to obtain the levels of respiratory complexes in the oral mucosa, and, thus, consider them in the study profile of children with ASD (Figure 3).

### 3.4. The Presence of Ala16val-SOD2 Polymorphism in Chilean Children with ASD

**Figure 4:**
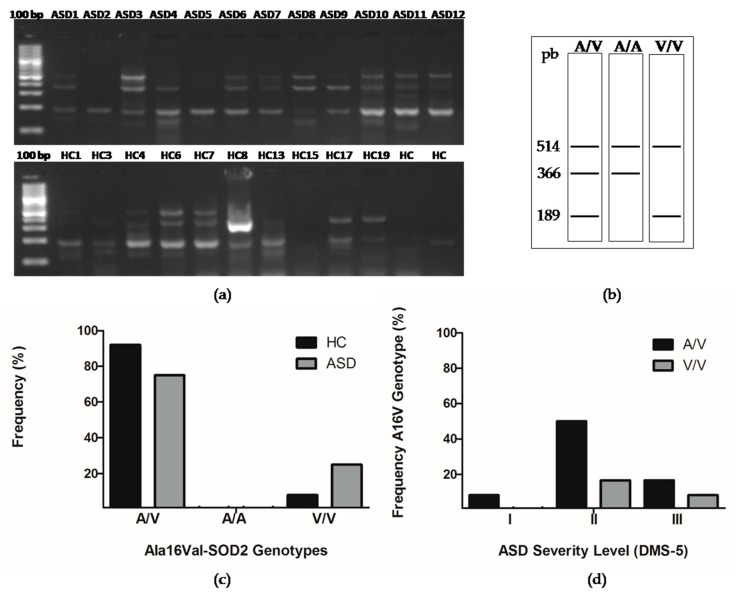
The presence of the Ala16val-superoxide dismutase (Ala16val-SOD2) polymorphism in Chilean children with ASD and in HCs. (**a**) Representative genotypes analyzed in an agarose gel electrophoresis. (**b**) Scheme of genotypes. (**c**) The observed genotypes were analyzed with a Chi-squared (X^2^) test with an alpha <0.05 statistical significance, obtaining a *P* value = 0.2231 n/s (non-significant). (**d**) The distribution of Ala16val-SOD2 polymorphism in the ASD severity level was analyzed with a Chi-squared (X^2^) test with an alpha <0.05 statistical significance, obtaining a *P* value = 0.1897 n/s (non-significant).

The single nucleotide polymorphism C47T at the 6q25 locus of the gene encoding for SOD2 results in the substitution of the Ala16Val amino acid and in the partial retention of SOD2 in the mitochondrial intermembrane space, reducing enzymatic activity and increasing oxidative stress [19]. However, the variability of the results obtained by the different research groups who have studied how this polymorphism may affect SOD2 activity indicates that it may be due to differences in diet, environmental exposure, and additional genetic factors [40,41,42,43,44,45]. In this study, we analyzed the presence of Ala16Val-SOD2 polymorphism in Chilean children (Figure 4a,b). Seventy-five percent of the population of children with ASD (9 of 12) and 92% of the population of children with HC (11 of 12), could be identified as being heterozygous for the Ala16Val-SOD2 (A/V) polymorphism. No HC or ASD children were homozygous for the Ala16 (A/A) allele. 

Regarding the Val16 allele (V/V), only one HC child was homozygous for it, and three children with ASD presented the polymorphism in homozygosis. When performing a Chi-square test, we found that these differences were not significant (Figure 4b,c). However, the number of children tested with ASD and HC is not enough to infer results. We were interested in analyzing the correlation of the A16VSOD2 polymorphism with the ASD severity levels. We observed that six of 12 (50%) of children with genotype A/V have a level II of severity, 17% (2 of 12) with genotype A/V have a level III severity, and 8% (1 of 12) present a level I of severity. On the other hand, 17% (2 of 12) with the V/V genotype presented a level II of severity and 8% (1 of 12) with a level III of severity. When performing a Chi-square test, we found that these differences were not significant (Figure 4d).

### 3.5. Expression of the Genes Encoding for HIGD2A, SOD2, DRP1, FIS1, MFN1, MFN2 and OPA1 from the Oral Mucosa of Chilean Children with ASD

The SOD2 enzyme is a metalloenzyme containing manganese. It is the only mitochondrial enzyme that dismutate the superoxide anion to H_2_O_2_ and is very important in the antioxidant defense of the eukaryotic cells [12,18]. Alterations in SOD2 enzyme function have been associated with different neurobiological disorders as well as with autism [12,18].

The deficit of SOD2 results in a higher production of free radicals, which cause damage to mitochondrial activity [12]. Likewise, as oxidative stress progresses, energy production weakens, and there is an increase in excitotoxicity in autistic children, which damages different macromolecules and genetic factors. Among them, an increase in the peroxidation of lipids, higher protein, and DNA oxidation can be noted [12,46,47].

**Figure 5:**
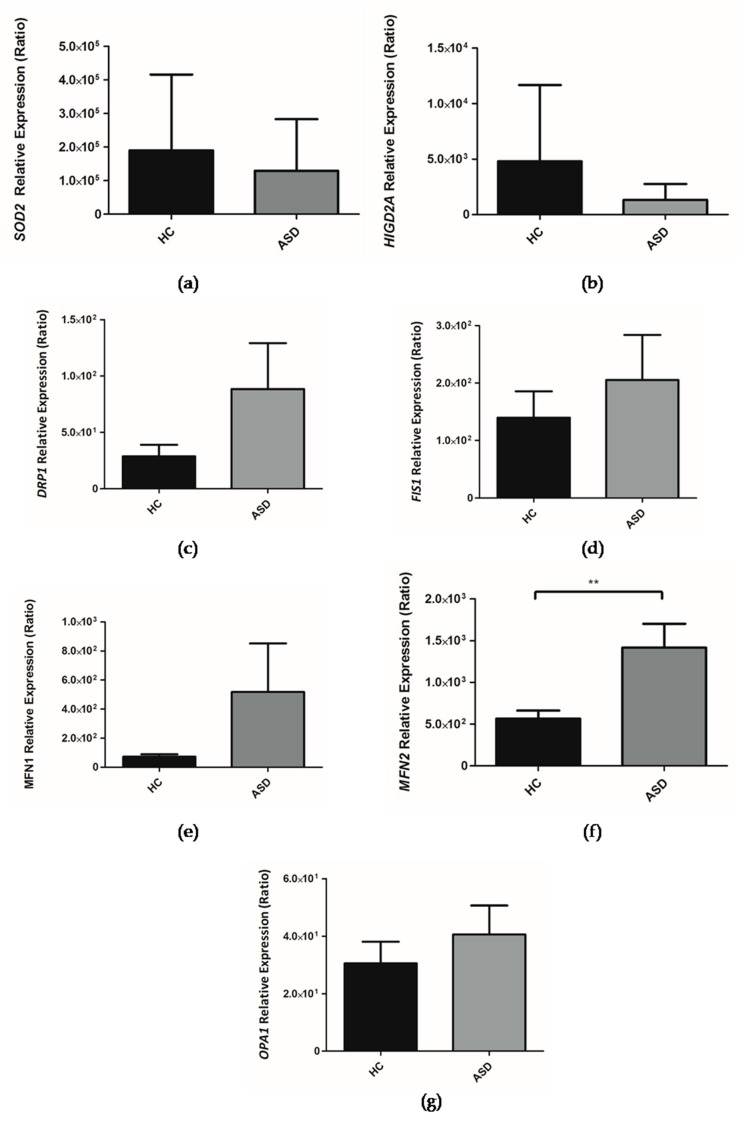
Relative expression of genes in children with Autism Spectrum Disorder (ASD) and Healthy Controls (HC). (**a**) *SOD2*, (**b**) *HIGD2A*, (**c**) *DRP1*, (**d**) *FIS1*, (**e**) *MFN1*, (**f**) *MFN2*, (**g**) *OPA1*. The *PPIA* gene was taken as a reference during the qPCR procedure. Each bar graph represents the mean ± SEM, *n* = 10 ASD and 11 HC children, analyzed by unpaired t-test (** *P* < 0.05).

As an initial overview of the status of the SOD2 enzyme in Chilean children with ASD, we decided to analyze the expression of the gene that encodes for SOD2 in the oral mucosa through RT-qPCR. Our results indicate, a downward trend in the expression of the *SOD2* gene in Chilean children with ASD (Figure 5a), which suggests that there is no alteration in the transcription of *SOD2* in ASD. We analyzed the correlation between the expression of the *SOD2* gene and the Ala16Val-SOD2 polymorphism; however, no correlation was observed (HC: *R*^2^ = 0.0473; ASD: *R*^2^ = 0.0889), indicating that the polymorphism does not affect the transcription of the *SOD2* gene.

The mitochondrial protein HIG2A promotes cell survival during hypoxic conditions [48]. Furthermore, HIG2A mediates the assembly of the complexes of the electron transport chain in respiratory supercomplexes or respirasomes [31,49]. These respirasomes enhance the transfer of electrons, channel substrates, stabilize protein complexes, enhance enzymatic activity, and reduce oxidative stress [32]. 

Our research group has focused on understanding the role of HIG2A as it relates to mitochondrial physiology. So far it has been established that the *HIGD2A* gene—encoding for the HIG2A protein—is upregulated by hypoxia and by glucose availability. The *HIGD2A* gene exhibits a differential expression based on tissue and age [33]. The absence of *HIGD2A* alters mitochondrial dynamics [33]. Within the framework of this study, we evaluated the expression of *HIGD2A* in the oral mucosa of Chilean children with ASD. It is shown that there is a downward trend in the expression of the *HIGD2A* gene (Figure 5b), suggesting that the transcription of *HIGD2A* gene does not have a direct impact on the development of ASD.

The assessment of the genes involved in mitochondrial dynamics, such as *MFN1*, *MFN2*, *OPA1* (mitochondrial fusion) and *DRP1* and *FIS1* (mitochondrial fission) was performed in the oral mucosa of children with ASD. It is shown that there is an upward trend in the expression of the *MFN1*, *OPA1*, *DRP1* and *FIS1* genes in the oral mucosa in children with ASD (Figure 5c–e,g) while a significant increase in the expression of the *MFN2* gene (Figure 5f) was shown.

### 3.6. Prediction of the Association of the Analyzed Genes with the Genetic Bases of ASD

**Figure 6:**
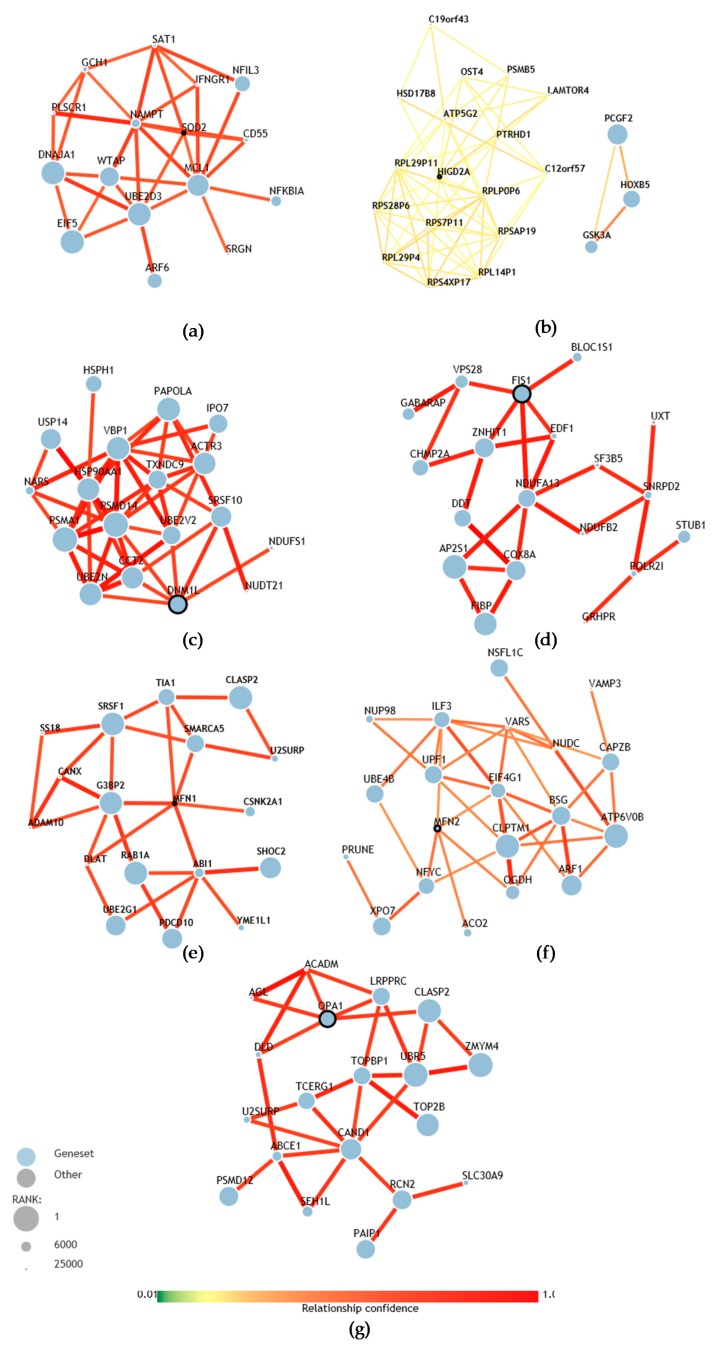
Prediction of gene association with the genetic bases of ASD. (**a**) Network of *SOD2* genetic associations, ranked #12001 among all genes in the human genome based on the prediction of association with ASD. (**b**) Network of *HIGD2A* genetic associations, rank #21384. (**c**) Network of *DRP1* (*DNM1L*) genetic associations, rank #2558. (**d**) Network of *FIS1* genetic associations, rank #2822. (**e**) Network of *MFN1* genetic associations, rank #22827. (**f**) Network of *MFN2* genetic associations, rank #11607. (**g**) Network of *OPA1* genetic associations, rank #3271 [50].

With the purpose of understanding the results of the gene expression reported above, we review the genetic associations of these genes with the computational tool (http://asd.princeton.edu) from the Lewis–Sigler Institute of Integrative Genomics at Princeton University (USA) that can predict the association of each of the genes of the human genome with the genetic basis of ASD. Among 25,825 genes, *SOD2* is located at position 12001, suggesting that the probability of association with ASD is low (Figure 6a) [50]. Upon analyzing the *HIGD2A* gene with the computational tool, we found that *HIGD2A* is ranked at position #21384 out of 25825, which suggests that the likelihood of association with ASD is very low (Figure 6b) [50]. 

The computational analysis of the genes involved in mitochondrial dynamics, such as *MFN1*, *MFN2*, *OPA1* (mitochondrial fusion) and *DRP1* and *FIS1* (mitochondrial fission) suggests a higher likelihood of association with the development of ASD. In particular, *OPA1* which is located at position #3271 of the ranking of genes associated with the development of ASD (Figure 6g), while *DRP1* is at position #2558 and *FIS1* at position #2822 (Figure 6c,d). In turn, these genes are related in the network with other genes that have a higher likelihood of association with the development of ASD [50]. *MFN1* is at position #22827 and *MFN2* is at position #11607. However, this last *MFN2* gene was the only one that showed a significantly higher expression in children with autism. When we review the networks of genetic associations of the *MFN2* gene (Table 2), the integration with genes that are more likely to be associated with the development of autism is evident.

The use of the computational tool from the Lewis–Sigler Institute for Integrative Genomics at the University of Princeton (USA) will be beneficial for future studies of different genes that might be involved in the development of ASD.

## 4. Discussion

The Chilean Association of Parents and Friends of the Autistic, ASPAUT, was one of the first centers created in Chile (1983) specialized in the treatment of persons with ASD, delivering pedagogical and therapeutic attention to achieve an integral development of persons with autism. Its Integral Diagnostic Team mentions that the diagnosis of ASD is delayed because families have the first complex experiences with children when they face the educational system and it is the pedagogues who refer them to suitable specialists (Table 1). Currently, only medical professionals (Neurologists and Child Psychiatrists) are responsible for this prescription and depend on their skills on the behavioral characteristics of Autism Spectrum Disorder. The generation of public policies on early detection is necessary for the timely identification of each child with ASD, and it is essential that the treatment of children begin at an early age to achieve a significant impact on their evolution. A study conducted in Chile by Hartmann et al. showed associations between ASD traits, physician support, community engagement, stigma, and family stress, highlighting “the importance of future research to better understand and treat Latin American children with ASD and their families” [51]. This study was an exploratory study carried out by a non-invasive sampling of the oral mucosa, which is a multi-stratified flat epithelial tissue that originates in the ectoderm, as does the nervous system [24]. 

In this study, we show that in the oral mucosa of Chilean children with ASD have a significant increase in mtDNA levels (Figure 1). These results agree with those obtained by the investigation of other groups that indicate an increase in mtDNA copies, and mtDNA deletions in children with ASD, which correlates with mitochondrial dysfunction [14,21]. However, other groups have not been able to identify differences in mtDNA levels in ASD [18,23]. The increase in mtDNA levels could account for a compensatory mechanism to strengthen the different mitochondrial functions that control cellular bioenergetics [14,21]. Furthermore, this condition is suggestive of alterations in the mitochondrial DNA replisome where different proteins are involved than those used for the replication of nuclear DNA, such as the TWINKLE helicase, the DNA polymerase γ, POLγ, and TFAM (Transcription Factor A, Mitochondrial), among others [38,39]. TFAM is an activator of mitochondrial transcription, it also functions as a histone-like in the packaging of mtDNA and as mtDNA levels follow TFAM levels [52,53].

An increase in the number of copies of mitochondrial DNA has been associated with good prognosis in glioma patients [54]. In addition, an improvement in survival in experimental conditions where human TFAM is overexpressed in an animal model of myocardial infarction [55]. The overexpression of human TFAM in mice diminishes the memory deterioration associated with aging [56]. The consequences of a high number of mtDNA copies in vivo are nucleoids enlargement, defects in transcription, accumulation of deletions in mtDNA, and deficiencies in the respiratory chain [57].

Several studies have reported oxidative stress in postmortem brain samples from patients with ASD, demonstrating oxidative damage to DNA, lipids, and proteins, and also reported alterations in the enzymatic activity of redox metabolism and lower concentrations of reduced glutathione (GSH) [12,58]. In this study, we evidenced higher protein carbonylation in the total protein of oral mucosa of ASD children (Figure 2). Furthermore, we show the presence of the respiratory complexes in the total protein of oral mucosa (Figure 3). Remarkably, Tang et al. (2013) in ASD postmortem brains in the BA21 temporal cortex, revealed significantly lower levels of respiratory complex I and decreased complex I and IV activities [18].

SOD2 is a metalloenzyme that contains manganese; it is the only enzyme in the mitochondria that converts superoxide to H_2_O_2_ [46,47], therefore, it has a fundamental role in dealing with the free radicals generated by the electron transport chain. In the gene coding for SOD2 (locus 6q25), the C47T single nucleotide polymorphism that results in the substitution of the amino acid Ala16Val has been studied extensively. Where the change from nucleotide cytosine (C) to thymidine (T) generates change in the amino acid sequence of SOD2 at the position of residue 16 of alanine (Ala) changes to valine (Val) occur. The Ala16Val-SOD2 polymorphism has been suggested to affect the transport of the SOD2 enzyme to the mitochondrial matrix as a result of modification to the mitochondrial targeting sequence. The 16Ala variant reaches the mitochondrial matrix, while the 16 Val variant is partially retained in the intermembrane space of the mitochondria. The presence of valine in position 16 reduces enzymatic activity and increases oxidative stress [19]. The altered cellular localization of SOD2 could be part of the neurodegeneration process that occurs in ASD. An exploratory study of the nutritional and metabolic biomarkers in ASD carried out by the Department of Integrative Medicine of the University of Kansas (USA), analyzed nine patients with ASD, of which four presented the Ala16Val polymorphism, three of them were homozygous (V/V) [20]. In our study three of twelve ASD children were homozygous (V/V) for the Ala16Val polymorphism while one of twelve HC children were homozygous (Figure 4). However, the number of children tested with ASD and HC is not enough to infer results. In addition to the exploration of Ala16Val-SOD2 polymorphism, we also studied the *SOD2* gene expression in the oral mucosa of ASD children, which shows a downward trend (Figure 5a). In autism postmortem brains, the Thalamus region-specific present alteration with *SOD2* gene shows upregulated expression [59]. While, in ASD postmortem brains in the BA21 temporal cortex presented a decreased mitochondrial antioxidant enzyme SOD2 [18].

Despite the known poor overall correlation between mRNAs and their protein products, differential mRNA expression studies implicitly assume that changes in mRNA expression have biological meaning, most likely mediated by corresponding changes in protein levels [60]. Transcript levels of *MFN1*, *MFN2*, *OPA1*, *DRP1*, and *FIS1* by themselves are not enough to predict MFN1, MFN2, OPA1, DRP1, and FIS1 protein levels in many scenarios and to, thus, explain genotype–phenotype relationships, however, high-quality data quantifying different levels of gene expression are necessary for the comprehensive understanding of MFN1, MFN2, OPA1, DRP1, and FIS1 biological processes in the development of ASD. In this study, there is an upward trend in the expression of the *MFN1*, *OPA1*, *DRP1,* and *FIS1* genes in the oral mucosa in children with ASD (Figure 5c–e,g) while a significant increase in the expression of the *MFN2* gene (Figure 5f) was shown. In autism postmortem brains, the Anterior Cingulate Gyrus region-specific *MFN2* gene shows downregulated expression [59]. The expression of *DRP1* (*DNML1*) was reduced in the Anterior Cingulate Gyrus and Motor Cortex region-specific alteration of autism patients [59]. Tang et al. (2013) show altered mitochondrial dynamics proteins in the brain of ASD patients, with decreased levels of mitochondrial fusion proteins; MFN1, MFN2, OPA1, and an increased level of mitochondrial fission proteins; DRP1 and FIS1 [18]. The treatment with butyrate (1 mM) of lymphoblastoid cell lines (LCL) derived from ASD patients increased the expression of genes involved in mitochondrial fission (*DRP1*, *FIS1*) [30]. Butyrate is a ubiquitous short-chain fatty acid principally derived from the enteric microbiome, and it is possible that the oral microbiome butyrate conditions the expression of the genes of the oral mucosal cells and this could explain the differences concerning that described in the brain.

## 5. Conclusions

There is a significant increase in mtDNA levels in ASD children in relation to HC children (healthy controls). 

The oral mucosa sample is a reliable tissue for assessing mtDNA levels in children with ASD. Furthermore, from this sample, it was possible to extract RNA for the evaluation of gene expression and proteins, which opens the door for new research possibilities in ASD or other diseases that are difficult to have access to or which make for risky blood samples.

Since the oral mucosa has an embryonic origin shared with that of the nervous system, it is a good alternative for exploratory studies on gene expression analysis associated with the development of ASD in humans. 

ASD is a complex neurodevelopmental disorder with a strong genetic basis. Despite this, only a small fraction of the genes that can be the cause of it are known and supported by strong genetic evidence from sequencing studies. This study constitutes a small contribution towards the understanding of the incidence of mitochondrial biology in the development of ASD.

## Figures and Tables

**Table 1:** Clinical data and demographic information of the children with Autism Spectrum Disorders who participated in this study.

Code	Gender	Diagnosis (Age Years)	DSM-IV	DSM-5 (Level)	Education ^1^	Therapy ^1^	Sample (Age Years)
ASD1	Male	ASD (3)	Severe	III ***	Pre-school	B ^2^, C ^3^, S ^4^	9
ASD2	Male	ASD (4)	Moderate	II **	Pre-school	B, C, S	9
ASD3	Male	ASD (3)	Moderate	II	Pre-school	B, C, S	8
ASD4	Male	ASD (4)	Moderate	II	Pre-school	B, C, S	8
ASD5	Male	ASD (3)	Moderate	II	Primary school	B, C, S	12
ASD6	Male	ASD (5)	Moderate	II	Primary school	B, C, S	11
ASD7	Male	ASD (1)	Moderate	II	Pre-school	B, C, S	7
ASD8	Male	ASD (2)	Mild	I *	Pre-school	B, C, S	7
ASD9	Female	ASD (6)	Moderate	II	Primary school	B, C, S	11
ASD10	Male	ASD (1)	Moderate	II	Primary school	B, C, S	9
ASD11	Male	ASD (2)	Severe	III	Primary school	B, C, S	12
ASD12	Male	ASD (2)	Severe	III	Primary school	B, C, S	12

^1^ Education and Therapy of children carried out in ASPAUT (Escuela Especial Magdalena Ávalos Cruz, San Miguel, Santiago, Chile); ^2^ B, Behavioral; ^3^ C, Communication; ^4^ S, Sensory. * Level I, restricted, repetitive behaviors, requiring support; ** Level II, social communication, requiring substantial support; *** Level III, severity level requiring very substantial support. DSM-IV (Diagnostic and Statistical Manual of Mental Disorders, fourth edition). DSM-5 (Diagnostic and Statistical Manual of Mental Disorders, fifth edition).

**Table 2:** Network of *MFN2* genetic associations in the human genome based on the prediction of association with ASD.

Gene	Gene Description	Chr ^1^	Edge Score	Rank ^2^
*NFYC*	Nuclear transcription factor Y, gamma	1p34.2	0.441	2752
*OGDH*	Oxoglutarate (alpha-ketoglutarate) dehydrogenase (lipoamide)	7p13	0.401	3680
*ACO2*	Aconitase 2, mitochondrial	22q13.2	0.395	7261
*EIF4G1*	Eukaryotic translation initiation factor 4 gamma, 1	3q27.1	0.352	3374
*ILF3*	Interleukin enhancer binding factor 3, 90 kDa	19p13.2	0.351	2897
*CLPTM1*	Cleft lip and palate associated transmembrane protein 1	19q13.32	0.329	355
*VAMP3*	Vesicle-associated membrane protein 3	1p36.23	0.328	24,000
*ARF1*	ADP-ribosylation factor 1	1q42.13	0.326	1225
*UBE4B*	Ubiquitination factor E4B	1p36.22	0.322	2086
*UPF1*	UPF1 regulator of nonsense transcripts homolog (yeast)	19p13.11	0.318	2170
*CAPZB*	Capping protein (actin filament) muscle Z-line, beta	1p36.13	0.316	2179
*NUDC*	NudC nuclear distribution protein	1p36.11	0.313	23,418
*XPO7*	Exportin 7	8p21.3	0.303	1922
*NUP98*	Nucleoporin 98 kDa	11p15.4	0.295	7869
*ATP6V0B*	ATPase, H+ transporting, lysosomal 21 kDa, V0 subunit b	1p34.1	0.294	277
*DLAT*	Dihydrolipoamide S-acetyltransferase	11q23.1	0.289	24,135
*BSG*	Basigin (Ok blood group)	19p13.3	0.289	1821
*VARS*	Valyl-tRNA synthetase	6p21.33	0.282	21,435
*NSFL1C*	NSFL1 (p97) cofactor (p47)	20p13	0.281	2031
*PRUNE*	Prune exopolyphosphatase	1q21.3	0.28	10,264

^1^ Chromosome Cytogenetic band. ^2^ Rank among all genes in the human genome based on the prediction of association with ASD.
